# Machine Learning-Driven Lung Sound Analysis: Novel Methodology for Asthma Diagnosis

**DOI:** 10.3390/arm93050032

**Published:** 2025-09-04

**Authors:** Ihsan Topaloglu, Gulfem Ozduygu, Cagri Atasoy, Guntug Batıhan, Damla Serce, Gulsah Inanc, Mutlu Onur Güçsav, Arif Metehan Yıldız, Turker Tuncer, Sengul Dogan, Prabal Datta Barua

**Affiliations:** 1 Department of Pulmonology, Faculty of Medicine, Kafkas University, 36000 Kars, Turkey; gulfemozduygu@gmail.com (G.O.); cagriatasoy92@gmail.com (C.A.); 2 Department of Thoracic Surgery, Faculty of Medicine, Kafkas University, 36000 Kars, Turkey; gbatihan@hotmail.com; 3 Department of Pulmonology, Faculty of Medicine, Health Sciences University, Izmir City Hospital, 35620 Izmir, Turkey; sercedamla@gmail.com; 4 Faculty of Medicine, Kafkas University, 36000 Kars, Turkey; drgulsahinanc1@gmail.com; 5 Department of Pulmonology, Faculty of Medicine, Bakırçay University, 35665 Izmir, Turkey; mutluonur.gucsav@bakircay.edu.tr; 6 Department of Computer Engineering, Faculty of Engineering, Ardahan University, 75000 Ardahan, Turkey; ametehanyildiz@ardahan.edu.tr (A.M.Y.); turkertuncer@firat.edu.tr (T.T.); 7 Department of Digital Forensics Engineering, College of Technology, Firat University, 23119 Elazig, Turkey; sdogan@firat.edu.tr; 8 School of Business (Information System), University of Southern Queensland, Toowoomba, QLD 4350, Australia; prabal.barua@usq.edu.au

**Keywords:** asthma, machine learning, respiratory sounds

## Abstract

**Highlights:**

**What are the main findings?**

- MFCC–TQWT features with ReliefF selection achieve 99.86% asthma classification.
- Quadratic SVM and Narrow Neural Network exceed 99% sensitivity and specificity.
- Non-invasive lung sound analysis diagnoses asthma in patients with normal spirometry.
- Lightweight (~14 kB) models enable real-time digital stethoscope analysis.

**What is the implication of the main finding?**

- External validation on two datasets confirms >98% accuracy and robustness.

**Abstract:**

Introduction: Asthma is a chronic airway inflammatory disease characterized by variable airflow limitation and intermittent symptoms. In well-controlled asthma, auscultation and spirometry often appear normal, making diagnosis challenging. Moreover, bronchial provocation tests carry a risk of inducing acute bronchoconstriction. This study aimed to develop a non-invasive, objective, and reproducible diagnostic method using machine learning-based lung sound analysis for the early detection of asthma, even during stable periods. Methods: We designed a machine learning algorithm to classify controlled asthma patients and healthy individuals using respiratory sounds recorded with a digital stethoscope. We enrolled 120 participants (60 asthmatic, 60 healthy). Controlled asthma was defined according to Global Initiative for Asthma (GINA) criteria and was supported by normal spirometry, no pathological auscultation findings, and no exacerbations in the past three months. A total of 3600 respiratory sound segments (each 3 s long) were obtained by dividing 90 s recordings from 120 participants (60 asthmatic, 60 healthy) into non-overlapping clips. The samples were analyzed using Mel-Frequency Cepstral Coefficients (MFCCs) and Tunable Q-Factor Wavelet Transform (TQWT). Significant features selected with ReliefF were used to train Quadratic Support Vector Machine (SVM) and Narrow Neural Network (NNN) models. Results: In 120 participants, pulmonary function test (PFT) results in the asthma group showed lower FEV1 (86.9 ± 5.7%) and FEV1/FVC ratios (86.1 ± 8.8%) compared to controls, but remained within normal ranges. Quadratic SVM achieved 99.86% accuracy, correctly classifying 99.44% of controls and 99.89% of asthma cases. Narrow Neural Network achieved 99.63% accuracy. Sensitivity, specificity, and F1-scores exceeded 99%. Conclusion: This machine learning-based algorithm provides accurate asthma diagnosis, even in patients with normal spirometry and clinical findings, offering a non-invasive and efficient diagnostic tool.

## 1. Introduction

Asthma, a chronic respiratory disease affecting millions worldwide, requires precise and timely diagnosis for effective management [1,2]. In stable asthma, auscultation, spirometry, and arterial blood gas results often appear normal between exacerbations [3]. Furthermore, spirometry outcomes depend on patient–technician cooperation, which may lead to missed diagnoses. Bronchial provocation tests are effective in detecting airway hyperresponsiveness; however, they have risks such as severe bronchoconstriction, which limits their widespread usage [4,5]. These challenges highlight the need for objective methods to reliably identify asthma without symptom dependence.

Recent advancements in biomedical signal processing and artificial intelligence (AI) have enabled the development of innovative diagnostic tools for respiratory medicine. Lung sound analysis has emerged as a non-invasive technique for assessing respiratory conditions by detecting signs of bronchial obstruction, such as wheezes and changes in airflow patterns [6]. Lung sounds, produced by airflow, encode critical information on airway patency, obstruction, and pathological changes. Digital stethoscopes combined with advanced signal processing enable the extraction of clinically relevant respiratory sound features, improving diagnosis [7].

Digital auscultation with modern signal processing can reveal acoustic signatures of airway narrowing. Spectral features and time–frequency representations can capture subtle, clinically relevant variations in lung sounds [8,9]. When paired with careful feature selection and standard machine learning models, these representations can support objective classification in respiratory disease [10].

The objective of this study was to propose a machine learning-based breath sound analysis approach as a non-invasive method to detect subtle airway changes, enabling the objective diagnosis of stable asthma despite normal spirometric findings. This approach aims to contribute to early and safe diagnostic processes by reducing the dependence on symptom-based assessments.

## 2. Materials and Methods

### 2.1. Study Population

A total of 120 participants were enrolled—60 patients with controlled asthma and 60 healthy controls—at the Pulmonology Outpatient Clinic of Kafkas University between February 2023 and December 2024. The study complied with the Declaration of Helsinki and was approved by the local ethics committee (Ethics No. 2023-233; 2 January 2023). All participants provided written informed consent before enrollment.

Patients with controlled asthma had a prior physician diagnosis based on clinical history and a positive bronchodilator reversibility test. Inclusion criteria were meeting GINA control criteria at enrollment; normal spirometry (forced expiratory volume in 1 s (FEV1)/forced vital capacity (FVC) ratio > 0.7 and FEV1 ≥ 80% predicted), with spirometry performed according to ATS/ERS standards; no abnormal findings on lung auscultation; no exacerbation in the prior 3 months; and age 18–65 years [11].

All patients with controlled asthma included in the study met the criteria for controlled asthma and were receiving their regular controller medications, including inhaled corticosteroids as prescribed. Medication use was recorded, but treatment regimens were not modified for the purposes of this study. Healthy participants were included if they had no history of respiratory or other chronic illness, demonstrated normal spirometry, and showed no pathological findings on lung auscultation [12].

Participants were excluded if they had a respiratory infection in the past four weeks, were active smokers or had a smoking history of ≥10 pack-years, or had significant comorbidities (e.g., cardiovascular, neuromuscular, metabolic disorders) affecting respiratory sounds. Individuals with chest wall abnormalities (kyphoscoliosis, pectus excavatum), BMI > 30, or occupational exposure to dust, chemicals, or air pollution were also excluded. Participants who could not undergo auscultation due to severe dyspnea, cognitive impairment, or recent thoracic surgeries were excluded to ensure reliable respiratory sound analysis.

No a priori power calculation was performed; instead, a balanced cohort of 120 participants (60 asthmatic, 60 healthy) was used, considered adequate in the light of the relevant literature [6,8,13].

### 2.2. Data Collection

Respiratory sound recordings were collected in a sound-treated room using the Littmann CORE Digital Stethoscope (3M, St. Paul, MN, USA) under standardized conditions to minimize ambient noise. Auscultation was performed bilaterally at six predefined points on the posterior thorax: the interscapular region (1st–3rd ribs), mid-scapular region (4th–6th ribs), and infrascapular region (7th–9th ribs). At each point, 15 s were recorded, yielding 90 s of recordings per participant. Participants were instructed to avoid caffeine and strenuous activity for 24 h before recording, and recordings were conducted in the morning (9:00–11:00 a.m.) to minimize circadian and fatigue-related variations.

Respiratory sounds were collected from six predefined anatomical regions bilaterally on the posterior thorax and analyzed as a single dataset. This approach was based on the assumption that all regions contribute equally to the diagnostic process of asthma. Additionally, to ensure the robustness and generalizability of the classification model, an external dataset was incorporated into the analysis. The combined dataset provided diverse respiratory sound patterns, allowing for a comprehensive evaluation of the model’s performance across different data sources. Each dataset was processed uniformly to maintain consistency in feature extraction and classification.

The 90 s of respiratory sound data per participant were segmented into 3 s clips, producing 30 recordings per participant. With 120 participants (60 patients with controlled asthma and 60 healthy controls), a total of 3600 respiratory sound recordings were generated. Each 3 s segment was treated as an independent sample for analysis, providing sufficient resolution for feature extraction and classification.

A 10-fold cross-validation method was used to validate the machine learning models. This technique divides the dataset into 10 equal parts, with each part serving as a test set once, while the remaining 90% of the data is used for training. For each fold, 3240 segments were used for training, and 360 segments were used for testing. This iterative process reduced overfitting and provided a reliable estimate of model performance across different subsets of the dataset.

Several strategies were implemented throughout the study to reduce potential bias:

- Homogeneous Groups: Age, sex, and BMI distributions were comparable between the asthma and control groups (*p* > 0.05).
- Technical Standardization: All recordings were obtained using the same digital stethoscope model and fixed chest sites.
- Segmentation: All recordings were divided into segments of equal length (3 s), and the analysis pipeline was automated.
- Quality Control: Noisy or distorted segments were identified using spectral analysis methods and excluded from the analysis.

### 2.3. Feature Extraction Layer

Briefly, 90-s respiratory sound recordings were divided into 3-s non-overlapping segments. Overlapping or windowing techniques were not applied. The 3 s duration was chosen to capture at least one full respiratory cycle while maintaining a manageable data size for feature extraction and model training.

The feature extraction process aimed to convert raw respiratory sound signals into structured data suitable for machine learning models. Two complementary techniques, MFCC and TQWT, were used to analyze spectral and time–frequency variations in respiratory sounds.

### 2.4. MFCC for Feature Extraction

In this study, MFCC was used to extract spectral features from respiratory sound signals. The MFCC technique, widely employed in audio signal processing, was adapted to analyze short-term temporal and spectral variations in the recordings.

Respiratory sound signals were segmented into non-overlapping 3 s blocks to ensure consistency across recordings, and each block was further divided into frames of 20–40 milliseconds to capture short-term temporal variations critical for identifying subtle respiratory pattern changes associated with pathological conditions. Fast Fourier Transform (FFT) converted the time-domain signal to its frequency representation. The magnitude spectrum was then mapped onto the Mel-frequency scale, emphasizing clinically relevant low-frequency components. To mimic the human perception of sound intensity and enhance sensitivity to small variations, the amplitude spectrum was transformed into a logarithmic scale. A Discrete Cosine Transform (DCT) was subsequently applied to the logarithmic amplitude values, compressing the data into compact and decorrelated coefficients (MFCCs) by reducing redundancy while preserving essential spectral features. Finally, the mean values of the MFCC coefficients were calculated for each frame to minimize variability and noise, ensuring that the extracted features robustly represented the respiratory sound patterns for machine learning analysis. The MFCC processing steps and equations are presented in Table 1.

### 2.5. TQWT for Feature Extraction

TQWT-based energy features were used for detailed time–frequency analysis by decomposing respiratory audio into sub-bands. This transform relies on three parameters: the quality factor Q (frequency selectivity/oscillation), the redundancy (oversampling and sub-band overlap), and the number of levels J (depth of decomposition). These jointly determine sub-band center frequencies and bandwidths, enabling analysis of frequency-specific components.

The process involved sub-band decomposition, where signals were divided into frequency-specific sub-bands using convolution operations. Energy computation quantified the energy in each sub-band, while statistical summarization (e.g., mean and variance) described sub-band characteristics. Shannon entropy measured signal irregularity, reflecting the complexity of respiratory sounds, and the maximum amplitude highlighted peak signal variations. Finally, the frequency band calculation determined the center frequency of each sub-band using TQWT parameters, enabling the identification of subtle acoustic patterns critical for distinguishing pathological conditions. TQWT energy approach effectively exploited both the temporal and spectral characteristics inherent within the signal, enabling a comprehensive analysis. The TQWT processing steps and equations are presented in Table 2.

### 2.6. Feature Combination and Selection

The features extracted from MFCC and TQWT methods were integrated into a unified feature vector, referred to as “combined features”. Each 3 s segment was represented by a 15-dimensional feature vector; stacking all segments produced a 3600 × 15 feature matrix (samples × features). By combining features from both methods, we maximized the representation of temporal and spectral characteristics in the dataset. The resulting feature matrix served as the input for classification models, preserving the key information required for accurate respiratory sound classification.

TQWT sub-bands: These emphasize low-frequency components (e.g., wheezing or stridor) caused by pathologies such as airway narrowing and bronchial wall thickening. Increased airflow turbulence, especially in asthmatic patients, alters the energy distribution in these bands.

MFCC coefficients: They summarize the spectral envelope of respiratory sounds. In asthma, attenuation or redistribution of higher-frequency components associated with small-airway inflammation can produce characteristic changes in MFCCs.

The combination of TQWT and MFCC features captures acoustic patterns consistent with asthma pathophysiology:

- Sub-band energy (TQWT) can reflect changes consistent with increased airway resistance.
- Specific MFCCs may index attenuation of higher-frequency content, potentially influenced by airway inflammation or mucus.

To optimize the feature set and enhance model efficiency, we employed the ReliefF algorithm to estimate feature relevance using nearest hits and misses in feature space. The algorithm assigns weights to each feature, prioritizing those that contribute most to differentiating between classes. In this algorithm, k = 10 was determined, and the first 10 most meaningful features were selected and given as input to the classifier.

The feature selection process using ReliefF reduced redundancy and focused on the most discriminative features, ensuring the machine learning models operated with optimal efficiency. This step was intended to improve the performance of the classification models by minimizing irrelevant or noisy features and enhancing the generalizability of the analysis.

The classification models, including Support Vector Machines (SVMs) and Neural Networks, were evaluated with stratified 10-fold cross-validation at the participant level (as described above) to reduce overfitting and avoid segment-level leakage.

### 2.7. Classification and Evaluation Process

**Figure 1:**
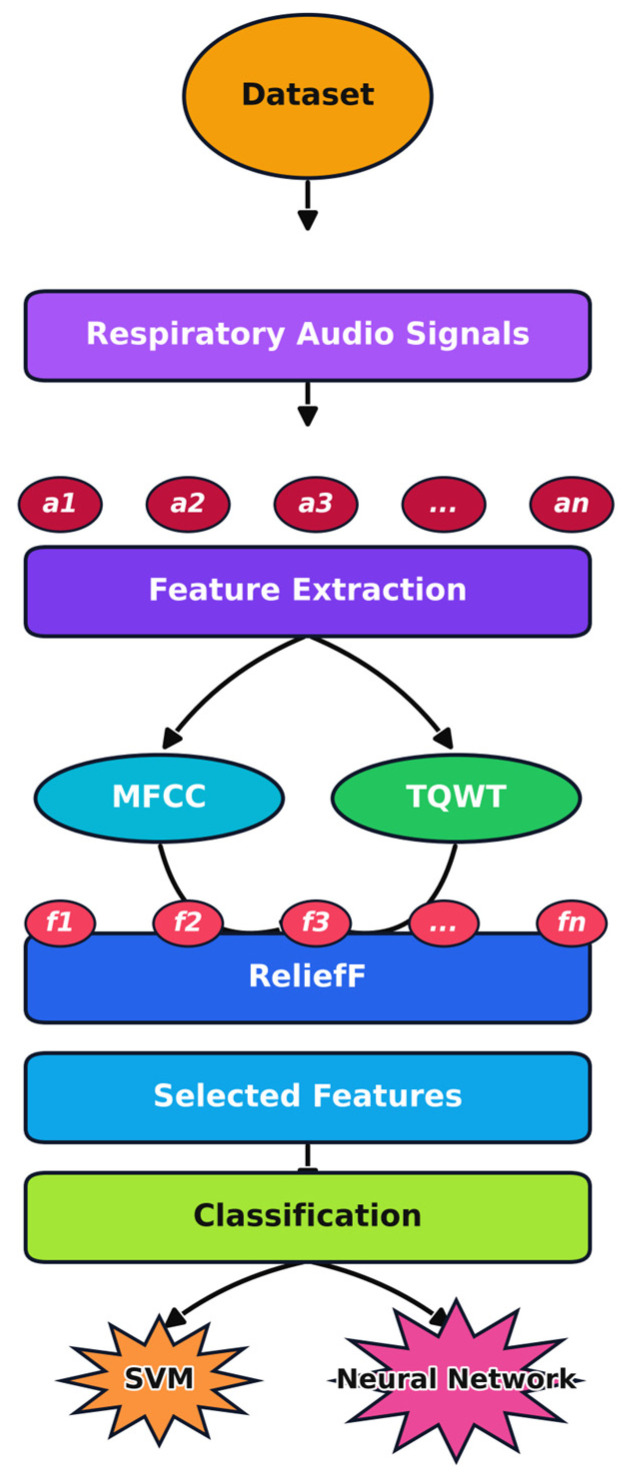
Workflow of the proposed respiratory sound classification model. (a1 … an: audio signal segments, f1 … fn: obtained features, MFCC: Mel-Frequency Cepstral Coefficients, ReliefF: feature selection algorithm, SVM: Support Vector Machine, TQWT: Tunable Q-factor Wavelet Transform).

In this study, machine learning models were trained and evaluated to distinguish patients with controlled asthma from healthy individuals based on respiratory sound analysis. The workflow included preprocessing (cleaning) and segmenting respiratory sound, extracting features using MFCC and TQWT pipelines, combining these features into a unified feature matrix, selecting the most informative features with the ReliefF algorithm, and training and evaluating classifiers (Figure 1).

The classification performance was assessed using commonly applied metrics such as accuracy, sensitivity, specificity, precision, and F1-score. These metrics are widely used for balanced datasets and provide a comprehensive evaluation of model performance. The hyperparameters for Quadratic SVM and NNN are given in Table 3.

### 2.8. Statistical Analysis

All statistical analyses were performed using IBM SPSS Statistics version 25.0 (IBM Corp., Armonk, NY, USA) and MATLAB R2023a (MathWorks, Natick, MA, USA). The normality of continuous variables was assessed using the Shapiro–Wilk test. Data are presented as mean ± standard deviation (SD) for normally distributed variables and as median (interquartile range) for non-normally distributed variables. Group comparisons between asthmatic and control participants were conducted using the independent samples *t*-test for normally distributed data and the Mann–Whitney U test for non-normally distributed data. Categorical variables were compared using the chi-square test or Fisher’s exact test, as appropriate. The performance of machine learning models was evaluated using accuracy, sensitivity, specificity, precision, and F1-score, calculated from confusion matrices. A two-tailed *p*-value < 0.05 was considered statistically significant.

## 3. Results

### 3.1. Patient Demographics and Pulmonary Function Test Results

This study included 120 participants: 60 patients with controlled asthma and 60 healthy controls. The mean age was 33 ± 7.7 years in the asthma group and 34.4 ± 10.7 years in the control group (*p* = 0.41). Gender distribution was 22 males and 38 females in the asthma group and 28 males and 32 females in the control group (*p* = 0.35). The mean body mass index (BMI) was 24.2 ± 2.9 kg/m^2^ in the asthma group and 23.1 ± 3.4 kg/m^2^ in the control group, with no statistically significant difference (*p* = 0.070). Pulmonary function test (PFT) results showed no significant differences between the two groups. Forced expiratory volume in one second (FEV1) was 86.9 ± 5.7% in the asthma group and 92.5 ± 10.8% in the control group (*p* = 0.001). The FEV1/FVC ratio was 86.1 ± 8.8% in the asthma group and 90.3 ± 11.4% in the control group (*p* = 0.026). Although pulmonary function test results were lower in the asthma group, they were within the normal range for controlled asthma.

### 3.2. Feature Extraction and Model Performance

**Figure 2:**
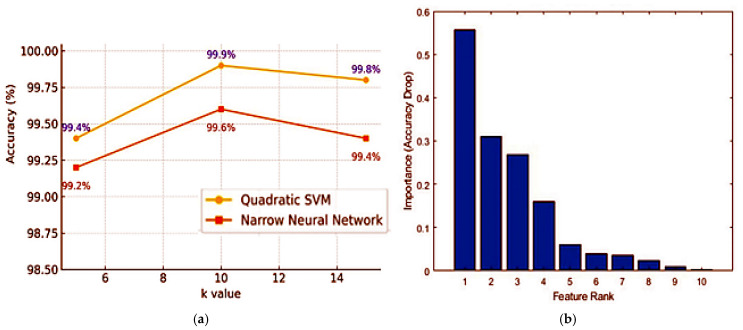
(**a**) Cross-validated accuracy versus the number of top ReliefF features (k) for Quadratic SVM and Narrow Neural Network; accuracy plateaus beyond k ≈ 10. (**b**) Permutation feature importance (accuracy drop) for the final model (k = 10).

Respiratory sound signals were processed using MFCC and TQWT techniques to extract 15 features per segment. ReliefF-based feature selection identified the ten most informative features from 3600 recordings (controlled asthma, *n* = 1800; healthy controls, n = 1800). Figure 2a shows cross-validated accuracy as a function of the number of top-k ReliefF features; accuracy peaks at k ≈ 10 and changes by ≤0.2 percentage points thereafter, indicating a practical plateau. Accordingly, k = 10 was fixed for the final model. Figure 2b presents permutation feature importance for the final model with k = 10 (mean accuracy drop after permuting each feature). ReliefF-based selection reduced redundancy and noise, enabling the models to operate more efficiently and with better generalizability.

**Figure 3:**
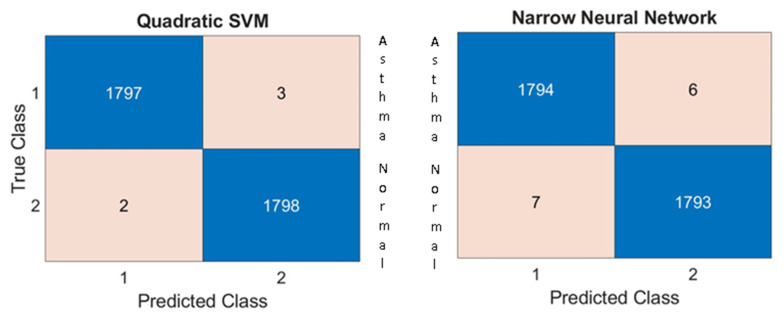
Confusion matrix for classification results.

Both classifiers demonstrated high performance. The quadratic SVM achieved an accuracy of 99.86%, and the narrow NN achieved 99.63%, averaged across stratified 10-fold participant-level cross-validation. Fold-aggregated confusion matrices are shown in Figure 3. Detailed metrics (accuracy, sensitivity/recall, specificity, precision, and F1-score) are summarized in Table 4.

**Figure 4:**
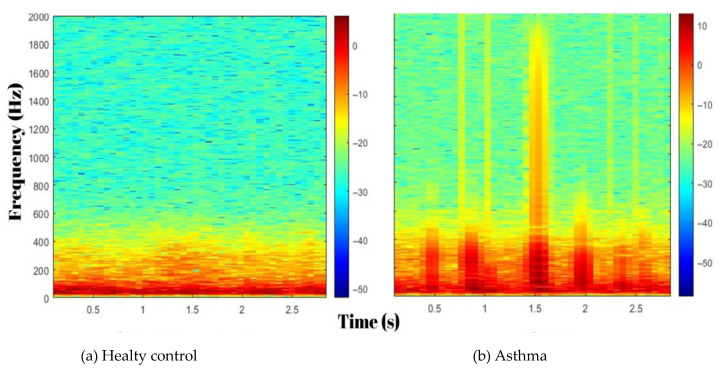
Heatmap of healthy controls and asthmatic individuals.

Figure 4 illustrates a heatmap of selected MFCC and TQWT features across healthy controls and patients with controlled asthma. Red indicates higher values in the asthma group (e.g., increased energy in specific TQWT sub-bands), and blue indicates higher values in controls (e.g., higher-frequency MFCC components). Concentration of TQWT energy in select bands and flattening of the MFCC envelope in asthma support physiologic plausibility (frequency- and entropy-based characteristics).

Training performance metrics for the Quadratic SVM classifier are summarized in Table 5. This table shows that the Quadratic SVM achieved an accuracy of 99.86%, precision of 99.88%, sensitivity of 99.83%, specificity of 99.89%, and F1-score of 99.86%, with an exceptionally low error rate of 0.1%. Its compact model size (~14 kB) and high prediction speed (~29,000 observations/s) enable efficient real-time implementation on portable devices. The Narrow Neural Network also demonstrated similarly high performance, with accuracy, precision, sensitivity, specificity, and F1-score all above 99.6%, confirming the robustness and generalizability of both models for respiratory sound classification. Table 5 reports training-time model characteristics. For the Quadratic SVM, accuracy was 99.86%, precision 99.88%, sensitivity 99.83%, specificity 99.89%, and F1-score 99.86% (fold means). The estimated error rate was 0.1%. The model’s compact size (~14 kB) and high prediction speed (~29,000 observations/s) support real-time deployment on portable devices. The narrow NN showed similarly high metrics (all ≥ 99.6%), supporting robustness and generalizability.

**Figure 5:**
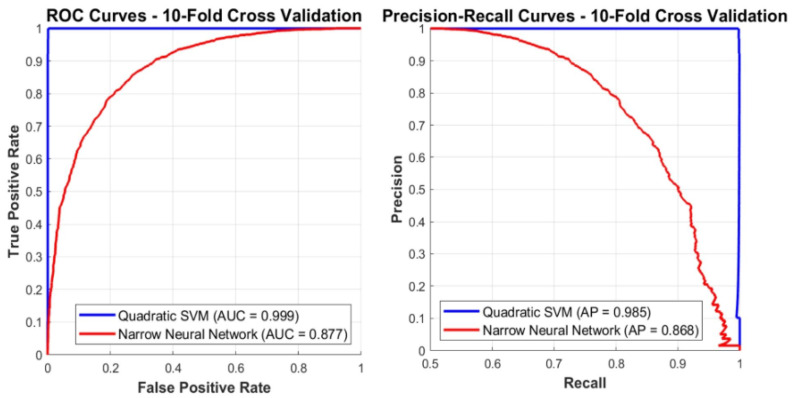
ROC and PR curves with AUC values.

The ROC-AUC and PR curves for these classification results are shown in Figure 5.

According to the graphs, the SVM classifier consistently outperforms the Neural Network (NN) classifier in both ROC-AUC and PR-AUC metrics and has a more robust and clearly defined decision boundary. Although the ROC-AUC metric is known for its strength, particularly in cases of imbalanced data distribution, it has been included in the results in order to provide a comprehensive evaluation of the classification performance of the model developed in this study [14]. In particular, the superiority of SVM in PR-AUC indicates higher reliability in positive class detection. Although NN achieves high accuracy, its lower AUC scores compared to SVM imply less distinct class separation. This limitation may stem from the network’s architecture and hyperparameters.

### 3.3. External Validation

**Figure 6:**
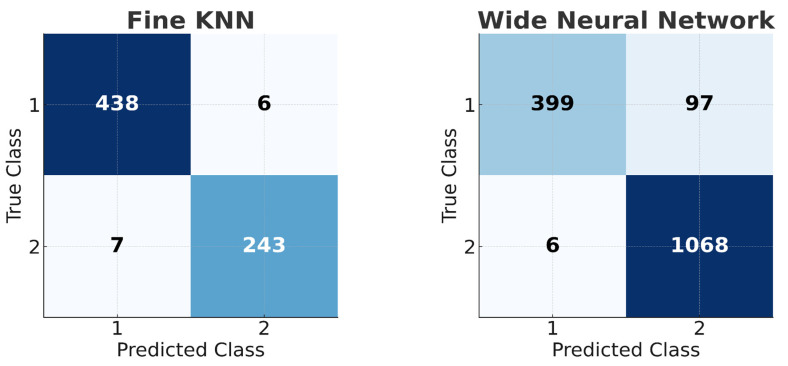
Confusion matrices for external validation (class labels: asthma = 1; healthy = 2).

External validation results are shown in Figure 6, which presents confusion matrices for the International Conference on Biomedical and Health Informatics 2017 (ICBHI 2017) dataset [15] and the Topaloglu et al. (2023) dataset [16] (class labels: asthma = 1, healthy = 2). In the ICBHI 2017 dataset, the proposed method achieved 98.3% accuracy with the Fine kNN classifier, while the Topaloglu et al. dataset yielded a 93.4% accuracy with the Wide Neural Network classifier. These results confirm the robustness of the proposed methodology across different datasets. Comparative performance with reference studies is summarized in Table 6.

## 4. Discussion

The rapid advancements in digital health technologies and machine learning have paved the way for novel approaches in respiratory diagnostics. Our study highlights the potential of machine learning algorithms with digital stethoscope technology to achieve highly accurate, non-invasive asthma diagnosis. The need for objective diagnostic methods is particularly crucial for patients with controlled (stable) asthma, as conventional tools such as spirometry and auscultation often fail to detect subtle airway changes during asymptomatic periods [17,18]. By employing MFCC and TQWT techniques for feature extraction and the ReliefF algorithm for feature selection, we achieved classification accuracies of 99.86% and 99.63% with Quadratic SVM and Narrow Neural Network models, respectively. In the optimization phase, adding a single feature derived from TQWT energy to the existing 14 MFCC features resulted in a substantial performance enhancement. The classification accuracy increased from 98.7% to 99.86% under the same cross-validation protocol. This improvement was achieved by subsequently employing the Relieff feature selection algorithm to identify the 10 most significant features among the combined set. These findings emphasize the feasibility of using respiratory sound analysis for reliable and scalable diagnostics, offering an innovative contribution to both medical and machine learning literature.

The Global Initiative for Asthma (GINA) 2024 guidelines recommend a combination of clinical assessment, spirometry, and bronchial provocation tests for diagnosing asthma [5]. However, in well-controlled asthma patients, particularly between exacerbations, spirometry results may appear normal, causing delays in diagnosis. Even in patients with normal lung function, mucosal inflammatory changes in the airways are present [19,20]. These changes may result in abnormal respiratory sounds that are undetectable by the human ear.

A study of 17 male patients by Henk J.W. Schreur et al. showed that the generation and/or transmission of lung sounds in asymptomatic, stable asthmatic patients with normal spirometric measurements differed from those in healthy subjects, even when lung function tests were within normal ranges [21]. Consistent with the literature, our findings showed that pulmonary function test results, such as FEV1 and FEV1/FVC ratios, were mildly reduced in the controlled asthma group compared to healthy controls but remained within the normal range for controlled asthma.

Bronchial provocation tests, another diagnostic tool, while valuable for identifying airway hyperresponsiveness, carry risks such as severe bronchoconstriction, limiting their widespread applicability. Additionally, these tests are resource-intensive procedures that may impact patient comfort [22]. Therefore, there is a need for risk-free, repeatable, and easily applicable diagnostic methods for patients with stable asthma. In this context, digital lung sound analysis has the potential to overcome the limitations of traditional diagnostic tools, offering significant benefits in terms of patient safety and diagnostic accuracy. As reported by Pramono et al., digital lung sound analysis has the capability to detect airway inflammation and early airway narrowing through acoustic signals, even in the absence of spirometric abnormalities [13].

Shimoda et al. demonstrated that lung sound analysis (LSA) effectively identifies airway inflammation in asthmatic patients by analyzing acoustic parameters and correlating them with clinical markers such as FEV1/FVC and FeNO levels [23]. Unlike Shimoda et al.’s reliance on specific clinical markers, our study highlights the ability of machine learning models to distinguish asthma patients from healthy controls with minimal dependence on clinical variables. This highlights the scalability and adaptability of our methodology, offering a complementary yet distinct perspective in advancing non-invasive asthma diagnostics.

In our study, MFCC and TQWT techniques were employed as complementary feature extraction methods for respiratory sound analysis, yielding strong classification performance. While MFCC excels at capturing spectral features, TQWT enhances the dataset with its time–frequency resolution capabilities. By integrating these two methods and optimizing feature selection with the ReliefF algorithm, we achieved high classification accuracies. Similarly, Chang G.C. et al. reported that using MFCC, they were able to distinguish asthma-related wheezes from other nonspecific sounds with 96.8% accuracy [24].

The methodology used in our study builds upon the experiences and findings of our previous research. For example, in one of our prior studies, we proposed an explainable attention-enhanced Residual Neural Network 18 (ResNet18)-based model for asthma detection using lung sounds recorded with a stethoscope. This model integrated attention mechanisms into ResNet18 to emphasize key features associated with asthma and enhance the interpretability of deep learning methods [16]. We take this experience a step further by providing a comprehensive analysis on a larger dataset using a machine learning model that achieves high accuracy rates.

Standardized data collection procedures with digital stethoscopes further enhance the clinical applicability of the findings. In similar studies in the literature (e.g., ICBHI 2017 dataset), deep learning models have reported accuracies of 95–98% accuracy on small datasets [25,26]. In contrast, the 99.86% accuracy achieved with SVM/NNN in this study demonstrates the advantage of traditional methods in low-data scenarios. However, considering the power of deep learning in capturing time–frequency patterns, it is recommended to test Transformer-based models using large multicenter datasets (e.g., >10,000 samples) in future studies. With a hybrid approach, integrating MFCC/TQWT features into Convolutional Neural Network (CNN)–Transformer architectures may allow learning both local and global acoustic features. Additionally, certain limitations must be acknowledged. The dataset was collected at a single center, and external testing used independent datasets, which may limit the generalizability of the results. However, in addition to the primary analysis, external validation was performed using two independent datasets to assess the generalizability of the proposed methodology. The first dataset utilized in this research is the publicly available ICBHI 2017 database. In its entirety, this comprehensive corpus consists of 5.5 h of respiratory sound recordings from 126 individuals with diverse ages, genders, and respiratory conditions, encompassing 920 annotated respiratory cycles. The recordings were acquired using digital stethoscopes and an AKG C417L microphone at variable sampling rates from 4 kHz to 44.1 kHz. For the present study, however, the analysis focused exclusively on a subset of recordings from 47 asthmatic and 27 healthy subjects within the database. This selection yielded a heterogeneous collection of 444 asthma and 250 control samples, which were derived from 47 asthmatic and 27 healthy control participants. Subsequently, these selected samples were partitioned into 3 s segments for analysis, yielding a 98.3% accuracy with the Fine k-Nearest Neighbors (kNN) classifier (Figure 6, confusion matrix). External testing in this dataset was restricted to the asthma and healthy control groups. As a second external validation, the dataset from Topaloglu et al. (2023) [16], comprising 496 asthmatic and 1074 control samples from 95 asthmatic and 108 healthy control participants, segmented into 3 s segments, achieved a 93.4% accuracy with the Wide Neural Network classifier (Figure 6, confusion matrix). These external validation results confirm the robustness and generalizability of the proposed model across different datasets.

While the respiratory sounds collected from six different anatomical regions were analyzed collectively in this study, this approach assumes equal contribution from all regions to the asthma diagnosis. Future research will investigate the diagnostic contributions of individual anatomical regions to better understand their relevance in asthma classification. This choice mirrored routine clinical auscultation practice. Although the primary goal of this research was to evaluate the generalization performance of the classification model, the potential diagnostic value of region-specific respiratory sounds remains an important area for further investigation. Incorporating external datasets strengthened the assessment of robustness and applicability. Future research could focus on analyzing regional differences in respiratory sounds and exploring how they influence diagnostic accuracy, particularly in varied clinical populations. This could provide deeper insights into the pathophysiological changes associated with asthma and refine the model’s applicability to specific patient profiles.

Future studies should aim to validate these findings in larger patient populations and expand datasets to include varying severities of asthma and other respiratory diseases. Additionally, exploring multimodal approaches that combine acoustic data with clinical and physiological parameters could provide a more comprehensive understanding of respiratory pathophysiology.

### Strengths and Limitations

MFCC–TQWT features with ReliefF selection, classified by SVM and a Narrow Neural Network, distinguish asthma from healthy individuals even when spirometry is normal. Performance was high under 10-fold cross-validation on 3600 three-second segments and was replicated on two independent external datasets, indicating robustness and generalizability. The pipeline is non-invasive, fast, and repeatable; its ~14 kB model and ~29,000 observations/s inference rate enable real-time, on-device use in point-of-care and home settings. The extracted acoustic patterns align with respiratory pathophysiology and may provide an objective adjunct to physician assessment, reducing reliance on respiratory function tests and enabling earlier detection during asymptomatic periods.

Key limitations include single-center recruitment, aggregate analysis across recording sites, lack of phenotype stratification, and potential domain shift across devices and environments. Future work should pursue multicenter, device-agnostic validation, quantify region- and phenotype-specific diagnostic value, and evaluate longitudinal performance for monitoring and relapse prediction, to support wider clinical adoption.

External validation in this study was performed using two datasets:

1. A publicly available dataset from Kaggle:
  Musaed, M. T. (2022, May). Asthma Detection Dataset Version 2 (ICBHI 2017) [Data set]. Kaggle. Available from: https://www.kaggle.com/datasets/mohammedtawfikmusaed/asthma-detection-dataset-version-2 (accessed on 13 July 2025).
2. A dataset derived from a previously published study by Topaloglu et al. (2023) [16].

## Figures and Tables

**Table 1:** MFCC processing steps and equations.

Steps	Equation
Fast Fourier Transform (FFT)	$X_{k}=\sum_{n=0}^{N-1}y_{n}\;e^{-j\;(\frac{2\pi }{N})\;kn}$
Mel-Frequency Scale Conversion	$M(f)=2595\;\cdot \;{log}_{10}\;(1+X_{k}=\frac{f}{700})$
Logarithmic Amplitude Calculation	$s_{m}=\log\left(\sum_{k}{\left|X\left[k\right]\right|}^{2}\;\cdot \;H_{m}\left[k\right]\right)$
Spectral Compression (DCT)	$C(n)=\sum_{m=1}^{M}S_{m}\;cos(\frac{\pi \;n\;(m-0.5)}{M})$
Feature Averaging	$mean(C_{k})\;\equiv \;\frac{1}{F}\;\sum_{f=1}^{F}C_{k}$

*X*[*k*]: frequency component; *y*[*n*]: time-domain signal; N: total number of samples; *k*: frequency index; *M*(*f*): Mel-scale conversion; *f*: frequency in Hz; *Hm*[*k*]: Mel filter applied to the frequency spectrum; *C*(*n*): MFCC coefficient; *Sm*: log-amplitude of the Mel filter; *F*: number of frames; *Ck*: value of the k-th MFCC coefficient in a single frame; *M*: total number of Mel filters.

**Table 2:** TQWT energy processing steps and equations.

Steps	Equation
Sub-Band Decomposition	$Sj\left(t\right)=\sum s\left(t\right)\cdot \;hj\left(n\right).\;\emptyset \left(t-n\right)$ n
Scaling Function	$1t\phi j\left(t\right)=\psi \left(v\right)\sqrt{Qj}\cdot rQ$
Energy Computation	$E\_j\;=\sum \_n|{\left(s_{j}\left(n\right)\right)}^{2}$
Total Energy Computation	$E_{total}\;=\;\sum_{j=1}^{J}E_{j}$ *j* = 1
Statistical Features	$\mu \_j=\frac{1}{N\_j}\;\sum \;S\_j(n)$ σ_j^2=1N_j ∑ S_j(n)
Shannon Entropy	Hj=−∑ pj(n)logpj (n) n
Maximum Amplitude	$P_{j}=max\_\{n\}|s\_j(n)\;\;|$
Frequency Band Calculation	f_j=f_low+(f_high−f_low)/2^j

*Sj*(*t*): sub-band component; *s*(*t*): time-domain signal; *hj*(*n*): impulse response of the j-th sub-band filter; *ϕ*(*t*): scaling function; *ϕj*: adjustable scaling function; *Q*: quality factor; *r*: redundancy factor; *Ej*: energy of the *j-th* sub-band; *Etotal*: total energy; *J*: total number of sub-bands; *μj*: mean of the *j-th* sub-band; *σj2*: variance of the *j-th* sub-band; *Nj*: number of samples in the *j-th* sub-band; *Hj*: Shannon entropy of the *j-th* sub-band; *pj*(*n*): normalized amplitude of the *j-th* sub-band; *Pj*: maximum amplitude of the *j-th* sub-band; *fj*: center frequency of the *j-th* sub-band.

**Table 3:** The hyperparameters used for classifiers.

Hyperparameter	Narrow Neural Network	Quadratic SVM
Model Basis	Feedforward Neural Network	Support Vector Machine
Number of Hidden Layers	1	Not Applicable
Number of Neurons	10	Not Applicable
Activation Function	ReLU	Not Applicable
Kernel Function	Not Applicable	quadratic’
Polynomial Order	Not Applicable	2
** C Parameter (Box Constraint)	Not Applicable	1
Gamma (Kernel Scale)	Not Applicable	Not Applicable (Only for Gaussian kernel)
Optimizer	sgdm (SGD with Momentum)	Not Applicable
Learning Rate	0.01	Not Applicable
Max Epochs	30 (Can be 1000 in the app)	Not Applicable
Batch Size	128	Not Applicable

Note: ** C (Box Constraint) is the regularization parameter that controls the trade-off between maximizing the margin and minimizing classification error in SVM models.

**Table 4:** Confusion matrix of algorithms—performance of classifications.

Model	Accuracy (%)	Precision (%)	Sensitivity (%)	Specificity (%)	F1-Score (%)
Quadratic SVM	99.86	99.88	99.83	99.88	99.86
Narrow Neural Network	99.63	99.61	99.66	99.61	99.61

**Table 5:** Training performance metrics.

Metric	Value
Accuracy (%)	99.86
Total Cost	5
Error Rate (%)	0.1
Prediction Speed	~29,000 observations/s
Training Time (seconds)	55.47
Model Size (compact)	~14 kB

**Table 6:** Comparative results in the other dataset.

Model	Accuracy (%)	Precision (%)	Sensitivity (%)	Specificity (%)	F1-Score (%)
ICBIH2017	98.13	98.43	98.65	97.20	98.54
Topaloğlu et al.	93.44	98.52	80.44	99.44	88.57
Our model	99.86	99.88	99.83	99.88	99.86

## Data Availability

The datasets generated and analyzed during the current study are not publicly available due to institutional data protection regulations, but are available from the corresponding author upon reasonable request.
